# Utility of Intraoperative Frozen Sections during Thyroid Surgery

**DOI:** 10.1155/2013/496138

**Published:** 2013-01-21

**Authors:** Russel Kahmke, Walter T. Lee, Liana Puscas, Richard L. Scher, Michael J. Shealy, Warner M. Burch, Ramon M. Esclamado

**Affiliations:** ^1^Division of Otolaryngology-Head and Neck Surgery, Department of Surgery, Duke University Medical Center, Duke University, Durham, NC 27710, USA; ^2^Section of Otolaryngology-Head and Neck Surgery, Department of Surgery, Durham VA Medical Center, Durham, NC 27705, USA; ^3^Division of Pathology Clinical Services, Department of Pathology, Duke University Medical Center, Duke University, Durham, NC 27710, USA; ^4^Division of Endocrinology, Metabolism, and Nutrition, Department of Medicine, Duke University Medical Center, Duke University, Durham, NC 27710, USA

## Abstract

*Objective*. To describe the usefulness of intraoperative frozen section in the diagnosis and treatment of thyroid nodules where fine needle aspirate biopsies have evidence of follicular neoplasm. *Study Design*. Retrospective case series. *Methods*. All patients have a fine needle aspirate biopsy, an intraoperative frozen section, and final pathology performed on a thyroid nodule after initiation of the Bethesda System for Reporting Thyroid Cytopathology in 2009 at a single tertiary referral center. Sensitivity, specificity, positive predictive value, and negative predictive value are calculated in order to determine added benefit of frozen section to original fine needle aspirate data. *Results*. The sensitivity and specificity of the frozen section were 76.9% and 67.9%, respectively, while for the fine needle aspirate were 53.8% and 74.1%, respectively. The positive and negative predictive values for the fine needle aspirates were 25% and 90.9%, respectively, while for the frozen sections were 27.8% and 94.8%, respectively. There were no changes in the operative course as a consequence of the frozen sections. *Conclusion*. Our data does not support the clinical usefulness of intraoperative frozen section when the fine needle aspirate yields a Bethesda Criteria diagnosis of follicular neoplasm, suspicious for follicular neoplasm, or suspicious for malignancy at our institution.

## 1. Introduction

The incidence of thyroid cancer increased 2.4-fold from 3.6 to 8.7 per 100,000 Americans [1] over a thirty-year period ending in 2002. The annual incidence of palpable thyroid nodules in North America is 0.1% [2] and most of those under 1 cm cannot be detected by physical exam alone [3]. The introduction of high resolution ultrasound technology has increased our ability to diagnose thyroid nodules [4]. A patient of male gender, aged <20 years or >70 years, with a family history of medullary thyroid carcinoma (MTC) or multiple endocrine neoplasia (MEN), rapid nodular growth, a firm or fixed nodule, a history of head and neck irradiation, a nodule that is >4 cm or is partially cystic, or a compressive sensation should raise increased suspicion for thyroid carcinoma [5].

In 2009, the Bethesda Criteria for Reporting Thyroid Cytopathology were published to create a common language by which multidisciplinary teams could accurately discuss the diagnosis and implications of a fine needle aspiration (FNA) biopsy [6]. The six categories found within the Bethesda Criteria each implies a different malignancy risk (Table 1) and treatment approaches ranging from watchful waiting to total thyroidectomy with postoperative radioactive iodine.

FNA biopsies with a diagnosis of follicular neoplasm, suspicious for follicular neoplasm, or suspicious for malignancy carry a 15–75% chance of malignancy [6]. Unfortunately, it is difficult to distinguish between follicular adenoma and follicular carcinoma based solely on an FNA biopsy because histologic evidence of capsular and/or vascular invasion is required to determine malignancy for a follicular lesion [7]. Approximately 30% of these FNA biopsies showing follicular lesions prove to be malignant on histologic examination [8]. A diagnostic thyroid lobectomy provides the tissue necessary for a pathologic diagnosis.

In thyroid surgery, intraoperative FS is used to assist in further surgical treatment at this point in care, with good concordance rates between FNA results and FS in the setting of papillary thyroid carcinoma [9]. However, FS is usually insufficient to determine true capsular or vascular invasion and deferral to a final pathologic diagnosis is often necessary in the setting of a follicular lesion [10]. The use of intraoperative FS has long been debated secondary to concerns about increased cost and operative time without a true consensus [11]. 

The overall objective of this study was to describe the usefulness of intraoperative FS in the diagnosis and treatment of thyroid nodules where FNA biopsies have evidence of follicular neoplasm. This was achieved by (1) determining the sensitivity and specificity of FS with both FNA and final pathology, (2) assessing the added benefit of FS to information obtained from the FNA, and (3) calculating the positive predictive value (PPV) and negative predictive value (NPV) of FS as a tool for intraoperative decision making. 

## 2. Materials and Methods

This retrospective chart review was approved by the Duke University School of Medicine Institutional Review Board. The review was performed on patients who have been evaluated and treated for a thyroid nodule within the Duke University Health System after the initiation of the Bethesda System for Reporting Thyroid Cytopathology in 2009. Our inclusion criteria required that a fine needle aspiration biopsy and an operative procedure with intraoperative frozen section and final pathology be performed at our tertiary care center. Our exclusion criteria included an FNA biopsy diagnostic of malignancy (e.g., papillary carcinoma),  nondiagnostic/unsatisfactory FNA samples, FNA reports not in congruence with the Bethesda Criteria, and a history of irradiation to the head and neck. 

FNA and intraoperative FS results were compared with final pathology. An FNA specimen was considered “suggests negative” if it was reported as “benign”, “negative,” or of “undetermined significance.” An FNA specimen was “suggests positive” if it showed “follicular neoplasm”, “suspicious for follicular neoplasm,” or “suspicious for malignancy”. Reports showing “Hurthle cell” within the description were considered to be part of the “follicular neoplasm” category. An FS specimen was considered “suggests negative” if the pathologist reported “benign”, “adenoma”, “negative for malignancy”, “nodular hyperplasia”, or “goiter”. An FS specimen was considered “suggests positive” for reports of “follicular lesion”, “follicular neoplasm”, “suspicious for carcinoma/malignancy”, “atypical features”, or “defer”. Final pathology was deemed positive if malignancy was found, regardless of size (i.e., incidentally found microcarcinoma). Sensitivity, specificity, positive predictive value (PPV), and negative predictive value (NPV) were calculated.

## 3. Results and Discussion

A total of 93 patients met inclusion criteria with 72 females (77.4%) and 21 males (22.6%). Median age was 51.9 years with a range between 9 and 76. One patient had two suspicious lesions which were analyzed, resulting in 94 thyroid nodules available for statistical analysis. 

Table 2 shows the stratification of FNA results that were available for analysis. No patients had FNA reporting malignancy or  nondiagnostic/unsatisfactory per study design. There were 32 samples that were read as atypia or follicular lesion of undetermined significance and 26 samples read as follicular neoplasm or suspicious for follicular neoplasm. The rest were read as either benign or suspicious for malignancy (34 and 2 samples, resp.). Despite the 0–3% risk of malignancy for benign diagnostic category, there were additional indications for surgery including suspicious ultrasound findings, compressive symptoms, persistent growth, or even patient preference. There were four patients noted to have  nondiagnostic/insufficient FNA biopsies, one with a negative FS and positive final pathology (papillary carcinoma), two with FS suggesting positive although final pathology was negative, and one with both negative FS and final pathology. There were 2 patients who were diagnostic malignancy where their FS and final pathology were both positive (papillary carcinoma and metastatic melanoma). 

Table 3 shows each patient and the correlation between the FNA Bethesda Criteria, FS, and final pathology. The sensitivity and specificity of FS were 76.9% and 67.9%, respectively. This is compared to the sensitivity and specificity for FNA, which were 53.8% and 74.1%, respectively. The PPV and NPV for FS were 27.8% and 94.8%, respectively. In comparison, FNA demonstrated a PPV of 25% and NPV of 90.9%. There were no changes in operative course as a consequence of a FS result. 

Our study did not demonstrate added information when intraoperative FS was used in patients with an FNA of benign, follicular neoplasm, suspicious for follicular neoplasm, or suspicious for malignancy reading. With this information, patients can be scheduled for a thyroid lobectomy with the knowledge that a diagnosis of malignancy will not be obtained until final pathologic analysis is completed. If malignancy is determined, a completion thyroidectomy can then be scheduled. The time and expense of intraoperative FS and scheduling operating room time for a total thyroidectomy, when it is not initially indicated, can therefore be spared.

The initiation of the Bethesda Criteria for Reporting Thyroid Cytopathology has greatly improved our ability to have accurate and meaningful conversations with our patients about their thyroid disease. In our case series, the sensitivity of FS was 76.9% compared to 53.8% for FNA, which is lower than previously published data [9, 12, 13]. However, these publications were performed prior to the Bethesda Criteria and this may account for these differences. Even when evaluating only patients with a diagnosis of malignancy, Makay et al. observed FS to only have a 72% sensitivity [14]. The PPV of both the FS and FNA biopsy were 27.8% and 25%, respectively. The challenges of FNA interpretation are well known and documented. Chang and colleagues [13] showed that when there was discrepancy between the FNA and FS, the FS was shown to be more accurate (78.9% versus 21.1%). 

Our results indicate that the addition of an FS does not allow for a more accurate and predictive result. There were no instances within our series where the FS altered the clinical course (i.e., conversion to a total thyroidectomy). There were no false positive results of malignancy for our FSs while there were two false negatives, one with a papillary microcarcinoma and the other a follicular carcinoma.

Future directions for the diagnosis and treatment of thyroid nodules should include addressing highly variable language used among pathologists in regard to thyroid intraoperative FS reporting; diagnosis may benefit from a more uniform language akin to that instituted for FNA biopsies. A standardized language may assist pathologists in their assessment as well as helping surgeons make decisions while in the operative theatre. 

## 4. Conclusion

Our data does not support the clinical usefulness of FS for FNA biopsies with the diagnosis of follicular neoplasm, suspicious for follicular neoplasm, or suspicious for malignancy at our institution. As such, decision for completion thyroidectomy should be determined by the final pathology. This data is useful for both counseling patients with thyroid lesions and subsequent surgical planning.

## Figures and Tables

**Table 1:** Fine needle aspiration biopsy diagnostic categories, adapted from Cibas and Ali (The Bethesda System for Thyroid Cytopathology) [6].

Diagnostic category	Risk of malignancy (%)
Nondiagnostic or unsatisfactory	1–4
Benign	0–3
Atypia or follicular lesion of undetermined significance	5–15
Follicular neoplasm or suspicious for follicular neoplasm	15–30
Suspicious for malignancy	60–75
Malignant	97–99

**Table 2:** Stratification of fine needle aspiration biopsies according to Bethesda system.

Diagnostic category	Number of samples
Non-diagnostic or unsatisfactory	0
Benign	34
Atypia or follicular lesion of undetermined significance	32
Follicular neoplasm or suspicious for follicular neoplasm	26
Suspicious for malignancy	2
Malignant	0

**Table 3:** Fine needle aspiration (FNA) Bethesda Criteria and frozen section (FS) compared with final pathology.

FNA	Frozen section	Final pathology		
Benign	Negative	Negative	19	
		Positive	1	FVPC
	Positive	Negative	12	
		Positive	2	FVPC ×2
AUS/FLUS	Negative	Negative	19	
		Positive	1	Follicular ca
	Positive	Negative	10	
		Positive	3	Follicular ca, FVPC, micropapillary ca
FN/SFN	Negative	Negative	14	
		Positive	1	Micropapillary ca
	Positive	Negative	6	
		Positive	5	Papillary ca, micropapillary ca ×2, FVPC, Hurthle cell ca
SM	Negative	Negative	1	
		Positive	0	
	Positive	Negative	1	
		Positive	0	

AUS/FLUS: atypia/follicular lesion of undetermined significance; FN/SFN: follicular neoplasm or suspicious for follicular neoplasm; SM: suspicious for malignancy; FNA: fine needle aspiration biopsy; FVPC: follicular variant of papillary carcinoma; ca: carcinoma.
